# *tert*-Butyl 6-methyl-2-oxo-4-[4-(trifluoro­meth­oxy)anilino]cyclo­hex-3-ene-1-carboxyl­ate

**DOI:** 10.1107/S1600536810046969

**Published:** 2010-11-20

**Authors:** Mariano S. Alexander, Henry North, Kenneth R. Scott, Ray J. Butcher

**Affiliations:** a Department of Pharmaceutical Sciences, Howard University, 2300 4th Street NW, Washington, DC 20059, USA; b Department of Chemistry, Howard University, 525 College Street NW, Washington, DC 20059, USA

## Abstract

In the title compound, C_19_H_22_F_3_NO_4_, the dihedral angle between the benzene ring and the conjugated part of the enaminone ring is 42.5 (1)°. The ester substituent makes a dihedral angle of 81.3 (2)° with this latter moiety. The crystal structure is held together by strong N—H⋯O and weak C—H⋯O inter­molecular inter­actions. The enaminone ring is disordered over two orientations with relative occupancies of 0.794 (4) and 0.206 (4).

## Related literature

The title compound posseses significant anti­convulsant properties. For the anti­convulsant properties of enamino­nes, see: Edafiogho *et al.* (1992 ▶); Eddington *et al.* (2003 ▶); Scott *et al.* (1993 ▶, 1995 ▶).
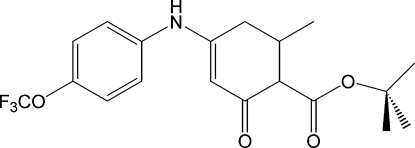


## Experimental

### 

#### Crystal data

C_19_H_22_F_3_NO_4_
*M* *_r_* = 385.38
Monoclinic, 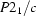

*a* = 13.7896 (3) Å
*b* = 12.0820 (2) Å
*c* = 11.0023 (2) Å
β = 91.1978 (18)°
*V* = 1832.65 (6) Å^3^
*Z* = 4
Cu *K*α radiation
μ = 1.01 mm^−1^
*T* = 123 K
0.48 × 0.18 × 0.08 mm

#### Data collection

Oxford Diffraction Xcalibur Ruby Gemini diffractometer
Absorption correction: multi-scan (*CrysAlis PRO*; Oxford Diffraction, 2007 ▶) *T*_min_ = 0.852, *T*_max_ = 1.000
7085 measured reflections
3607 independent reflections
3095 reflections with *I* > 2σ(*I*)
*R*_int_ = 0.018

#### Refinement

*R*[*F* ^2^ > 2σ(*F* ^2^)] = 0.058
*wR*(*F* ^2^) = 0.160
*S* = 1.06
3607 reflections
262 parameters
H-atom parameters constrained
Δρ_max_ = 0.66 e Å^−3^
Δρ_min_ = −0.39 e Å^−3^

### 

Data collection: *CrysAlis PRO* (Oxford Diffraction, 2007 ▶); cell refinement: *CrysAlis PRO*; data reduction: *CrysAlis PRO*; program(s) used to solve structure: *SHELXS97* (Sheldrick, 2008 ▶); program(s) used to refine structure: *SHELXL97* (Sheldrick, 2008 ▶); molecular graphics: *SHELXTL* (Sheldrick, 2008 ▶); software used to prepare material for publication: *SHELXTL*.

## Supplementary Material

Crystal structure: contains datablocks I, global. DOI: 10.1107/S1600536810046969/hg2750sup1.cif

Structure factors: contains datablocks I. DOI: 10.1107/S1600536810046969/hg2750Isup2.hkl

Additional supplementary materials:  crystallographic information; 3D view; checkCIF report

## Figures and Tables

**Table 1:** Hydrogen-bond geometry (Å, °)

*D*—H⋯*A*	*D*—H	H⋯*A*	*D*⋯*A*	*D*—H⋯*A*
N1—H1*A*⋯O2^i^	0.88	2.08	2.886 (2)	153
C2—H2*A*⋯O2^i^	0.95	2.58	3.333 (3)	136
C6—H6*A*⋯O3^ii^	0.95	2.55	3.385 (3)	147
C9*B*—H9*BA*⋯O3^iii^	0.99	2.44	3.40 (6)	162

Symmetry codes: (i) 
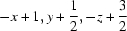
; (ii) 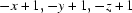; (iii) 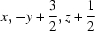.

**Fig. 1.**
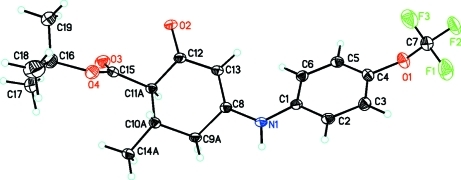
Diagram of tert-butyl-4-(4-trifluoromethoxyphenylamino)-6-methyl-2-oxocyclohex-3-en-1-oate showing atom labeling scheme. Thermal ellipsoids drawn at the 30% probability level.

**Fig. 2.**
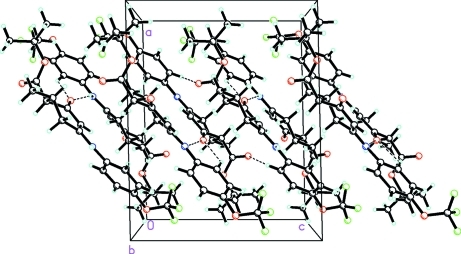
The molecular packing for tert-butyl-4-(4-trifluoromethoxyphenylamino)-6-methyl-2-oxocyclohex-3-en-1-oate viewed down the b axis. Intermolecular interactions are shown by dashed lines.

## supplementary crystallographic information

### Comment

Our research on enaminones has led to several compounds possessing anticonvulsant properties (Edafiogho *et al.*, 1992; Eddington *et al.*, 2003; Scott *et al.*, 1993, 1995). The present work is part of a structural study of enaminones. Our group has extensively studied the effects of modification of the enaminone with substitutions at the methyl ester, ethyl ester, and without the ester group. We synthesized a series of carbo-*tert*-butoxy esters to evaluate the effect of added bulk and lipophilicity to the ester functionality. The title compound, *tert*-butyl-4-(4-trifluoromethoxyphenylamino)-6-methyl-2-oxocyclohex-3-en-1-oate (10) is highly active, with activity at <100 mg kg^-1^.

The compound was exclusively active in maximal electroshock seizure evaluation (MES) in mice, indicative of protection against tonic-clonic convulsions in humans (1/4 rats were protected at 15 min then 3/4 rats at 2 h and 4 hrs at post dose 50 mg kg^-1^ in rats, orally). The MES test with mice revealed no activity in the 30 minute study, however in the 4 h MES test 1/1 animals were protected at 30 mg kg^-1^, 3/3 animals protected at 100 mg kg^-1^, and 1/1 at 300 mg kg^-1^ with no toxicity. The scMET (subcutaneous phentylenetetrazole assessment), indicative of protection against absence seizures was 0/2 animals protected in doses of 62.5 to 500 mg kg^-1^. The compound displayed no toxicity from 62.5 to 500 mg kg^-1^ from 15 min to 24 h time range in all doses. A four hour MES test showed 4/16 mice protected at 100 mg kg^-1^ dose and maximium protection (7/8 mice protected) at 150 and 200 mg kg^-1^. In mice, a MES ED_50_ (median effective dose) of 121.87 mg kg-1 and TD_50_ (median toxic dose) of >500 mg kg-1, provided a protective index PI (defined as the ration of the median toxic dose to the median effective dose) at 95% confidence interval.

In view of the therapeutic interest in this compound its structure was determined. The conformation adopted by the molecule is such that the dihedral angle between the phenyl ring and conjugated part of the enaminone ring is 42.5 (1)°. The ester substituent makes a dihedral angle of 81.3 (2)° with this latter moiety. The crystal structure is held together by strong N—H···O and weak C—H···O intermolecular interactions. The enaminone ring is disordered over two conformations with relative occupancies of 0.794 (4)/0.206 (4).

### Experimental

4-Carbo-t-butoxy-5-methylcyclohexane-1,3-dione (6.11 g, 27 mmol), mp 145–146°C (lit. mp 130–131.5°C), and 4-trifluoromethoxyaniline (4.428 ml g, 33 mmol) were added to a mixture of absolute EtOH (100 ml) and EtOAc (100 ml), and the solution was refluxed and stirred for 6 h with azeotropic removal of water by Dean-Stark trap. Evaporation under reduced pressure yielded a yellow solid which was recrystallized from 2-PrOH, 47% yield (mp 168–171°C). ).

### Refinement

H atoms were placed in geometrically idealized positions and constrained to ride on their parent atoms with a C—H distances of 0.95 to 1.00 Å *U*_iso_(H) = 1.2*U*_eq_(C) and 0.98 Å for CH_3_ [*U*_iso_(H) = 1.5*U*_eq_(C)]. The H atoms attached to N were idealized with an N–H distance of 0.88 Å. The enaminone ring is disordered over two conformations with relative occupancies of 0.794 (4)/0.206 (4). Each component was constrained to have similar metrical and thermal parameters

### Crystal data

**Table:**

C_19_H_22_F_3_NO_4_	*F*(000) = 808
*M**_r_* = 385.38	*D*_x_ = 1.397 Mg m^−^^3^
Monoclinic, *P*2_1_/*c*	Cu *K*α radiation, λ = 1.54184 Å
Hall symbol: -P 2ybc	Cell parameters from 4437 reflections
*a* = 13.7896 (3) Å	θ = 4.9–74.0°
*b* = 12.0820 (2) Å	µ = 1.00 mm^−^^1^
*c* = 11.0023 (2) Å	*T* = 123 K
β = 91.1978 (18)°	Needle plate, colorless
*V* = 1832.65 (6) Å^3^	0.48 × 0.18 × 0.08 mm
*Z* = 4	

### Data collection

**Table:**

Oxford Diffraction Xcalibur Ruby Gemini diffractometer	3607 independent reflections
Radiation source: Enhance (Cu) X-ray Source	3095 reflections with *I* > 2σ(*I*)
graphite	*R*_int_ = 0.018
Detector resolution: 10.5081 pixels mm^-1^	θ_max_ = 74.1°, θ_min_ = 4.9°
ω scans	*h* = −16→14
Absorption correction: multi-scan (*CrysAlis PRO*; Oxford Diffraction, 2007)	*k* = −14→8
*T*_min_ = 0.852, *T*_max_ = 1.000	*l* = −13→13
7085 measured reflections	

### Refinement

**Table:**

Refinement on *F*^2^	Primary atom site location: structure-invariant direct methods
Least-squares matrix: full	Secondary atom site location: difference Fourier map
*R*[*F*^2^ > 2σ(*F*^2^)] = 0.058	Hydrogen site location: inferred from neighbouring sites
*wR*(*F*^2^) = 0.160	H-atom parameters constrained
*S* = 1.06	*w* = 1/[σ^2^(*F*_o_^2^) + (0.0769*P*)^2^ + 1.5676*P*] where *P* = (*F*_o_^2^ + 2*F*_c_^2^)/3
3607 reflections	(Δ/σ)_max_ < 0.001
262 parameters	Δρ_max_ = 0.66 e Å^−^^3^
0 restraints	Δρ_min_ = −0.38 e Å^−^^3^

### Special details

**Table:**

Geometry. All e.s.d.'s (except the e.s.d. in the dihedral angle between two l.s. planes) are estimated using the full covariance matrix. The cell e.s.d.'s are taken into account individually in the estimation of e.s.d.'s in distances, angles and torsion angles; correlations between e.s.d.'s in cell parameters are only used when they are defined by crystal symmetry. An approximate (isotropic) treatment of cell e.s.d.'s is used for estimating e.s.d.'s involving l.s. planes.
Refinement. Refinement of *F*^2^ against ALL reflections. The weighted *R*-factor *wR* and goodness of fit *S* are based on *F*^2^, conventional *R*-factors *R* are based on *F*, with *F* set to zero for negative *F*^2^. The threshold expression of *F*^2^ > σ(*F*^2^) is used only for calculating *R*-factors(gt) *etc*. and is not relevant to the choice of reflections for refinement. *R*-factors based on *F*^2^ are statistically about twice as large as those based on *F*, and *R*- factors based on ALL data will be even larger.

### Fractional atomic coordinates and isotropic or equivalent isotropic displacement parameters (Å^2^)

**Table:**

	*x*	*y*	*z*	*U*_iso_*/*U*_eq_	Occ. (<1)
F1	0.10734 (15)	0.64674 (16)	1.23883 (16)	0.0709 (6)	
F2	0.04027 (13)	0.49236 (14)	1.20234 (15)	0.0589 (5)	
F3	0.19525 (14)	0.5113 (2)	1.1945 (2)	0.0850 (7)	
O1	0.09751 (12)	0.58259 (15)	1.05175 (15)	0.0420 (4)	
O2	0.59047 (12)	0.50627 (12)	0.62115 (16)	0.0397 (4)	
O3	0.66640 (13)	0.58950 (15)	0.36210 (19)	0.0490 (5)	
O4	0.75913 (13)	0.67009 (14)	0.50910 (16)	0.0437 (4)	
N1	0.38563 (13)	0.78606 (14)	0.78117 (17)	0.0301 (4)	
H1A	0.3844	0.8587	0.7870	0.036*	
C1	0.31707 (15)	0.72966 (17)	0.85070 (19)	0.0272 (4)	
C2	0.28473 (16)	0.78157 (17)	0.95577 (19)	0.0305 (5)	
H2A	0.3124	0.8503	0.9803	0.037*	
C3	0.21290 (16)	0.73416 (19)	1.0246 (2)	0.0336 (5)	
H3A	0.1906	0.7702	1.0955	0.040*	
C4	0.17405 (15)	0.63344 (18)	0.9886 (2)	0.0310 (5)	
C5	0.20450 (16)	0.58090 (18)	0.8851 (2)	0.0315 (5)	
H5A	0.1766	0.5121	0.8615	0.038*	
C6	0.27590 (15)	0.62856 (17)	0.81557 (19)	0.0295 (4)	
H6A	0.2969	0.5926	0.7440	0.035*	
C7	0.11132 (18)	0.5574 (2)	1.1683 (2)	0.0422 (6)	
C8	0.45375 (14)	0.74432 (16)	0.70599 (18)	0.0262 (4)	
C9A	0.5003 (12)	0.8322 (14)	0.6279 (13)	0.0279 (12)	0.794 (4)
H9AA	0.5105	0.8995	0.6778	0.033*	0.794 (4)
H9AB	0.4546	0.8515	0.5606	0.033*	0.794 (4)
C10A	0.5972 (2)	0.7986 (2)	0.5737 (3)	0.0267 (6)	0.794 (4)
H10A	0.6476	0.7949	0.6403	0.032*	0.794 (4)
C11A	0.58523 (18)	0.6829 (2)	0.5167 (2)	0.0256 (5)	0.794 (4)
H11A	0.5310	0.6865	0.4550	0.031*	0.794 (4)
C14A	0.6263 (10)	0.8853 (13)	0.4833 (13)	0.0344 (13)	0.794 (4)
H14A	0.6888	0.8651	0.4486	0.052*	0.794 (4)
H14B	0.6323	0.9571	0.5242	0.052*	0.794 (4)
H14C	0.5769	0.8901	0.4182	0.052*	0.794 (4)
C9B	0.505 (5)	0.834 (6)	0.641 (5)	0.0279 (12)	0.206 (4)
H9BA	0.5494	0.8727	0.6977	0.033*	0.206 (4)
H9BB	0.4569	0.8878	0.6086	0.033*	0.206 (4)
C10B	0.5616 (9)	0.7870 (10)	0.5372 (11)	0.0267 (6)	0.206 (4)
H10B	0.5148	0.7565	0.4752	0.032*	0.206 (4)
C11B	0.6240 (7)	0.6924 (8)	0.5856 (9)	0.0256 (5)	0.206 (4)
H11B	0.6718	0.7133	0.6513	0.031*	0.206 (4)
C14B	0.630 (4)	0.878 (5)	0.471 (6)	0.0344 (13)	0.206 (4)
H14D	0.6391	0.8572	0.3861	0.052*	0.206 (4)
H14E	0.6933	0.8818	0.5133	0.052*	0.206 (4)
H14F	0.5989	0.9513	0.4742	0.052*	0.206 (4)
C12	0.55529 (16)	0.60027 (17)	0.6181 (2)	0.0329 (5)	
C13	0.48038 (15)	0.63603 (16)	0.69731 (19)	0.0281 (4)	
H13A	0.4482	0.5826	0.7453	0.034*	
C15	0.67459 (18)	0.64028 (19)	0.4539 (3)	0.0421 (6)	
C16	0.85219 (18)	0.6464 (2)	0.4484 (2)	0.0421 (6)	
C17	0.8520 (2)	0.6974 (2)	0.3218 (3)	0.0543 (7)	
H17A	0.8062	0.6571	0.2687	0.081*	
H17B	0.9172	0.6927	0.2886	0.081*	
H17C	0.8323	0.7752	0.3267	0.081*	
C18	0.9252 (3)	0.7032 (3)	0.5303 (4)	0.0734 (10)	
H18A	0.9232	0.6706	0.6118	0.110*	
H18B	0.9099	0.7823	0.5350	0.110*	
H18C	0.9903	0.6937	0.4976	0.110*	
C19	0.86786 (19)	0.5226 (2)	0.4465 (2)	0.0435 (6)	
H19A	0.8613	0.4929	0.5288	0.065*	
H19B	0.9330	0.5065	0.4173	0.065*	
H19C	0.8195	0.4881	0.3921	0.065*	

### Atomic displacement parameters (Å^2^)

**Table:**

	*U*^11^	*U*^22^	*U*^33^	*U*^12^	*U*^13^	*U*^23^
F1	0.0923 (14)	0.0713 (12)	0.0501 (10)	−0.0257 (10)	0.0258 (9)	−0.0172 (9)
F2	0.0668 (10)	0.0592 (10)	0.0515 (9)	−0.0206 (8)	0.0218 (8)	0.0063 (8)
F3	0.0611 (11)	0.1082 (17)	0.0865 (14)	0.0289 (11)	0.0204 (10)	0.0574 (13)
O1	0.0411 (9)	0.0485 (10)	0.0367 (9)	−0.0135 (8)	0.0053 (7)	0.0018 (7)
O2	0.0395 (9)	0.0199 (7)	0.0601 (11)	0.0059 (6)	0.0104 (8)	0.0037 (7)
O3	0.0455 (10)	0.0339 (9)	0.0674 (12)	0.0013 (8)	−0.0016 (9)	−0.0065 (9)
O4	0.0535 (11)	0.0357 (9)	0.0425 (9)	0.0030 (8)	0.0121 (8)	−0.0088 (7)
N1	0.0375 (10)	0.0172 (8)	0.0358 (9)	0.0011 (7)	0.0060 (7)	−0.0013 (7)
C1	0.0280 (10)	0.0223 (9)	0.0315 (10)	0.0030 (8)	0.0007 (8)	0.0014 (8)
C2	0.0344 (11)	0.0230 (10)	0.0342 (11)	−0.0017 (8)	−0.0001 (9)	−0.0034 (8)
C3	0.0378 (12)	0.0333 (11)	0.0299 (10)	−0.0012 (9)	0.0034 (9)	−0.0049 (9)
C4	0.0299 (10)	0.0315 (11)	0.0316 (10)	−0.0039 (9)	0.0019 (8)	0.0039 (9)
C5	0.0331 (11)	0.0240 (10)	0.0372 (11)	−0.0014 (8)	−0.0021 (9)	−0.0015 (8)
C6	0.0335 (11)	0.0249 (10)	0.0301 (10)	0.0012 (8)	0.0008 (8)	−0.0031 (8)
C7	0.0416 (13)	0.0422 (13)	0.0432 (13)	0.0024 (11)	0.0114 (10)	0.0049 (11)
C8	0.0269 (10)	0.0222 (10)	0.0295 (10)	−0.0001 (8)	−0.0014 (8)	−0.0007 (8)
C9A	0.0309 (19)	0.0166 (10)	0.036 (3)	−0.0004 (13)	0.0004 (19)	0.000 (2)
C10A	0.0268 (16)	0.0212 (12)	0.0321 (16)	−0.0029 (11)	−0.0003 (11)	−0.0011 (11)
C11A	0.0264 (13)	0.0200 (11)	0.0303 (13)	−0.0010 (9)	−0.0018 (9)	−0.0006 (10)
C14A	0.0413 (17)	0.022 (2)	0.040 (3)	−0.0023 (16)	0.009 (2)	0.0008 (19)
C9B	0.0309 (19)	0.0166 (10)	0.036 (3)	−0.0004 (13)	0.0004 (19)	0.000 (2)
C10B	0.0268 (16)	0.0212 (12)	0.0321 (16)	−0.0029 (11)	−0.0003 (11)	−0.0011 (11)
C11B	0.0264 (13)	0.0200 (11)	0.0303 (13)	−0.0010 (9)	−0.0018 (9)	−0.0006 (10)
C14B	0.0413 (17)	0.022 (2)	0.040 (3)	−0.0023 (16)	0.009 (2)	0.0008 (19)
C12	0.0289 (10)	0.0196 (10)	0.0503 (13)	−0.0002 (8)	0.0050 (9)	0.0015 (9)
C13	0.0313 (10)	0.0191 (9)	0.0340 (10)	−0.0007 (8)	0.0020 (8)	0.0017 (8)
C15	0.0382 (13)	0.0247 (11)	0.0641 (17)	0.0021 (9)	0.0184 (11)	0.0109 (11)
C16	0.0371 (13)	0.0393 (13)	0.0499 (14)	0.0003 (10)	0.0023 (10)	−0.0053 (11)
C17	0.0510 (16)	0.0486 (15)	0.0641 (18)	0.0055 (13)	0.0220 (13)	0.0111 (13)
C18	0.064 (2)	0.0525 (18)	0.102 (3)	−0.0054 (16)	−0.0229 (19)	−0.0140 (18)
C19	0.0424 (13)	0.0411 (13)	0.0470 (14)	0.0055 (11)	0.0003 (10)	−0.0025 (11)

### Geometric parameters (Å, °)

**Table:**

F1—C7	1.331 (3)	C11A—C12	1.559 (3)
F2—C7	1.316 (3)	C11A—H11A	1.0000
F3—C7	1.311 (3)	C14A—H14A	0.9800
O1—C7	1.328 (3)	C14A—H14B	0.9800
O1—C4	1.416 (3)	C14A—H14C	0.9800
O2—C12	1.235 (3)	C9B—C10B	1.50 (7)
O3—C15	1.185 (3)	C9B—H9BA	0.9900
O4—C15	1.352 (3)	C9B—H9BB	0.9900
O4—C16	1.487 (3)	C10B—C11B	1.520 (15)
N1—C8	1.361 (3)	C10B—C14B	1.63 (7)
N1—C1	1.405 (3)	C10B—H10B	1.0000
N1—H1A	0.8800	C11B—C12	1.510 (10)
C1—C2	1.396 (3)	C11B—C15	1.740 (10)
C1—C6	1.398 (3)	C11B—H11B	1.0000
C2—C3	1.384 (3)	C14B—H14D	0.9800
C2—H2A	0.9500	C14B—H14E	0.9800
C3—C4	1.384 (3)	C14B—H14F	0.9800
C3—H3A	0.9500	C12—C13	1.432 (3)
C4—C5	1.377 (3)	C13—H13A	0.9500
C5—C6	1.385 (3)	C16—C18	1.503 (4)
C5—H5A	0.9500	C16—C19	1.512 (3)
C6—H6A	0.9500	C16—C17	1.523 (4)
C8—C13	1.363 (3)	C17—H17A	0.9800
C8—C9B	1.48 (7)	C17—H17B	0.9800
C8—C9A	1.516 (18)	C17—H17C	0.9800
C9A—C10A	1.530 (17)	C18—H18A	0.9800
C9A—H9AA	0.9900	C18—H18B	0.9800
C9A—H9AB	0.9900	C18—H18C	0.9800
C10A—C14A	1.504 (17)	C19—H19A	0.9800
C10A—C11A	1.540 (4)	C19—H19B	0.9800
C10A—H10A	1.0000	C19—H19C	0.9800
C11A—C15	1.515 (3)		
			
C7—O1—C4	118.68 (19)	H9BA—C9B—H9BB	108.1
C15—O4—C16	119.42 (19)	C9B—C10B—C11B	108 (2)
C8—N1—C1	129.19 (17)	C9B—C10B—C14B	113 (4)
C8—N1—H1A	115.4	C11B—C10B—C14B	110 (2)
C1—N1—H1A	115.4	C9B—C10B—H10B	108.4
C2—C1—C6	119.10 (19)	C11B—C10B—H10B	108.4
C2—C1—N1	117.59 (18)	C14B—C10B—H10B	108.4
C6—C1—N1	123.18 (19)	C12—C11B—C10B	106.5 (8)
C3—C2—C1	120.8 (2)	C12—C11B—C15	101.2 (6)
C3—C2—H2A	119.6	C10B—C11B—C15	102.4 (8)
C1—C2—H2A	119.6	C12—C11B—H11B	115.0
C2—C3—C4	119.0 (2)	C10B—C11B—H11B	115.0
C2—C3—H3A	120.5	C15—C11B—H11B	115.0
C4—C3—H3A	120.5	C10B—C14B—H14D	109.5
C5—C4—C3	121.3 (2)	C10B—C14B—H14E	109.5
C5—C4—O1	116.67 (19)	H14D—C14B—H14E	109.5
C3—C4—O1	122.0 (2)	C10B—C14B—H14F	109.5
C4—C5—C6	119.9 (2)	H14D—C14B—H14F	109.5
C4—C5—H5A	120.1	H14E—C14B—H14F	109.5
C6—C5—H5A	120.1	O2—C12—C13	123.3 (2)
C5—C6—C1	120.0 (2)	O2—C12—C11B	115.9 (4)
C5—C6—H6A	120.0	C13—C12—C11B	112.7 (4)
C1—C6—H6A	120.0	O2—C12—C11A	119.9 (2)
F3—C7—F2	110.1 (2)	C13—C12—C11A	116.59 (18)
F3—C7—O1	114.7 (2)	C11B—C12—C11A	35.1 (4)
F2—C7—O1	108.6 (2)	C8—C13—C12	122.08 (19)
F3—C7—F1	105.3 (3)	C8—C13—H13A	119.0
F2—C7—F1	106.2 (2)	C12—C13—H13A	119.0
O1—C7—F1	111.7 (2)	O3—C15—O4	125.9 (2)
N1—C8—C13	126.06 (19)	O3—C15—C11A	120.0 (2)
N1—C8—C9B	111 (2)	O4—C15—C11A	114.0 (2)
C13—C8—C9B	122 (2)	O3—C15—C11B	149.8 (4)
N1—C8—C9A	113.0 (6)	O4—C15—C11B	83.5 (4)
C13—C8—C9A	121.0 (6)	C11A—C15—C11B	32.1 (3)
C9B—C8—C9A	6(3)	O4—C16—C18	102.5 (2)
C8—C9A—C10A	114.8 (10)	O4—C16—C19	108.8 (2)
C8—C9A—H9AA	108.6	C18—C16—C19	111.5 (2)
C10A—C9A—H9AA	108.6	O4—C16—C17	110.3 (2)
C8—C9A—H9AB	108.6	C18—C16—C17	110.6 (3)
C10A—C9A—H9AB	108.6	C19—C16—C17	112.6 (2)
H9AA—C9A—H9AB	107.5	C16—C17—H17A	109.5
C14A—C10A—C9A	108.7 (8)	C16—C17—H17B	109.5
C14A—C10A—C11A	113.0 (5)	H17A—C17—H17B	109.5
C9A—C10A—C11A	108.2 (7)	C16—C17—H17C	109.5
C14A—C10A—H10A	108.9	H17A—C17—H17C	109.5
C9A—C10A—H10A	108.9	H17B—C17—H17C	109.5
C11A—C10A—H10A	108.9	C16—C18—H18A	109.5
C15—C11A—C10A	114.4 (2)	C16—C18—H18B	109.5
C15—C11A—C12	109.83 (19)	H18A—C18—H18B	109.5
C10A—C11A—C12	108.5 (2)	C16—C18—H18C	109.5
C15—C11A—H11A	108.0	H18A—C18—H18C	109.5
C10A—C11A—H11A	108.0	H18B—C18—H18C	109.5
C12—C11A—H11A	108.0	C16—C19—H19A	109.5
C8—C9B—C10B	111 (4)	C16—C19—H19B	109.5
C8—C9B—H9BA	109.5	H19A—C19—H19B	109.5
C10B—C9B—H9BA	109.5	C16—C19—H19C	109.5
C8—C9B—H9BB	109.5	H19A—C19—H19C	109.5
C10B—C9B—H9BB	109.5	H19B—C19—H19C	109.5
			
C8—N1—C1—C2	153.2 (2)	C14B—C10B—C11B—C15	−61 (2)
C8—N1—C1—C6	−31.0 (3)	C10B—C11B—C12—O2	157.3 (6)
C6—C1—C2—C3	0.0 (3)	C15—C11B—C12—O2	50.7 (6)
N1—C1—C2—C3	176.0 (2)	C10B—C11B—C12—C13	−52.9 (8)
C1—C2—C3—C4	0.7 (3)	C15—C11B—C12—C13	−159.6 (3)
C2—C3—C4—C5	−1.0 (3)	C10B—C11B—C12—C11A	51.5 (7)
C2—C3—C4—O1	−177.2 (2)	C15—C11B—C12—C11A	−55.2 (5)
C7—O1—C4—C5	123.9 (2)	C15—C11A—C12—O2	−14.0 (3)
C7—O1—C4—C3	−59.7 (3)	C10A—C11A—C12—O2	−139.7 (2)
C3—C4—C5—C6	0.5 (3)	C15—C11A—C12—C13	171.5 (2)
O1—C4—C5—C6	176.93 (19)	C10A—C11A—C12—C13	45.8 (3)
C4—C5—C6—C1	0.2 (3)	C15—C11A—C12—C11B	79.3 (7)
C2—C1—C6—C5	−0.4 (3)	C10A—C11A—C12—C11B	−46.4 (7)
N1—C1—C6—C5	−176.20 (19)	N1—C8—C13—C12	−177.9 (2)
C4—O1—C7—F3	−44.1 (3)	C9B—C8—C13—C12	−4(3)
C4—O1—C7—F2	−167.7 (2)	C9A—C8—C13—C12	2.3 (7)
C4—O1—C7—F1	75.5 (3)	O2—C12—C13—C8	169.1 (2)
C1—N1—C8—C13	−11.5 (4)	C11B—C12—C13—C8	22.0 (5)
C1—N1—C8—C9B	174 (2)	C11A—C12—C13—C8	−16.5 (3)
C1—N1—C8—C9A	168.4 (6)	C16—O4—C15—O3	4.9 (4)
N1—C8—C9A—C10A	161.2 (6)	C16—O4—C15—C11A	−172.1 (2)
C13—C8—C9A—C10A	−19.0 (11)	C16—O4—C15—C11B	177.6 (4)
C9B—C8—C9A—C10A	85 (30)	C10A—C11A—C15—O3	−141.6 (2)
C8—C9A—C10A—C14A	170.9 (8)	C12—C11A—C15—O3	96.1 (3)
C8—C9A—C10A—C11A	47.8 (9)	C10A—C11A—C15—O4	35.6 (3)
C14A—C10A—C11A—C15	56.9 (7)	C12—C11A—C15—O4	−86.7 (2)
C9A—C10A—C11A—C15	177.4 (6)	C10A—C11A—C15—C11B	55.1 (6)
C14A—C10A—C11A—C12	179.9 (6)	C12—C11A—C15—C11B	−67.2 (6)
C9A—C10A—C11A—C12	−59.6 (6)	C12—C11B—C15—O3	36.2 (11)
N1—C8—C9B—C10B	−166 (2)	C10B—C11B—C15—O3	−73.7 (10)
C13—C8—C9B—C10B	19 (4)	C12—C11B—C15—O4	−132.0 (5)
C9A—C8—C9B—C10B	−60 (28)	C10B—C11B—C15—O4	118.2 (7)
C8—C9B—C10B—C11B	−51 (4)	C12—C11B—C15—C11A	65.9 (6)
C8—C9B—C10B—C14B	−173 (3)	C10B—C11B—C15—C11A	−44.0 (6)
C9B—C10B—C11B—C12	69 (3)	C15—O4—C16—C18	173.6 (2)
C14B—C10B—C11B—C12	−167 (2)	C15—O4—C16—C19	−68.2 (3)
C9B—C10B—C11B—C15	174 (3)	C15—O4—C16—C17	55.8 (3)

### Hydrogen-bond geometry (Å, °)

**Table:**

*D*—H···*A*	*D*—H	H···*A*	*D*···*A*	*D*—H···*A*
N1—H1A···O2^i^	0.88	2.08	2.886 (2)	153
C2—H2A···O2^i^	0.95	2.58	3.333 (3)	136
C6—H6A···O3^ii^	0.95	2.55	3.385 (3)	147
C9B—H9BA···O3^iii^	0.99	2.44	3.40 (6)	162

Symmetry codes: (i) −*x*+1, *y*+1/2, −*z*+3/2; (ii) −*x*+1, −*y*+1, −*z*+1; (iii) *x*, −*y*+3/2, *z*+1/2.
